# Interaction between posaconazole and flucloxacillin in a lung transplant patient: decrease in plasma exposure of posaconazole and possible undertreatment of invasive aspergillosis: case report

**DOI:** 10.1186/s12890-022-01904-4

**Published:** 2022-03-27

**Authors:** Saartje Verfaillie, Laurent Godinas, Isabel Spriet, Robin Vos, Geert M. Verleden

**Affiliations:** 1 Department of Respiratory Diseases, Lung Transplantation Unit, University Hospital Gasthuisberg, Leuven, Belgium; 2 Department of Pharmaceutical and Pharmacological Sciences, University of Leuven, Leuven, Belgium; 3 Lung Transplantation Unit, University Hospitals Leuven, 49 Herestraat, 3000 Leuven, Belgium

**Keywords:** Case report, Lung transplantation, Azole, Posaconazole, Flucloxacillin

## Abstract

**Background:**

Variability in triazole plasma concentrations by drug interactions is well known. An interaction between voriconazole and flucloxacillin has already been described. In our case we observed a similar interaction between posaconazole and flucloxacillin, which in our knowledge has not ever been reported in literature.

**Case presentation:**

A 60-year-old male who had a double lung transplantation for end-stage chronic obstructive pulmonary disease was being treated with voriconazole for invasive pulmonary aspergillosis (IPA). During this treatment he presented at the emergency room and was diagnosed with endocarditis for which a combination of amoxicillin, flucloxacillin and gentamicin was initiated. A known interaction between voriconazole and flucloxacillin was observed, with a drop of the voriconazole levels, and treatment for IPA was switched to posaconazole. After ending the treatment for endocarditis, the patient had a catheter infection for which flucloxacillin was reinitiated. Unexpectedly we saw a similar immediate drop in posaconazole levels, recovering after ending treatment with flucloxacillin.

**Conclusions:**

We describe a new interaction between posaconazole and flucloxacillin. Presumably the underlying mechanism is activation of the pregnane X receptor by flucloxacillin, which can induce cytochrome P450, uridine glucuronosyl transferase (UGT1A4) and P-glycoprotein. We advise caution when combining flucloxacillin and triazoles, because interactions may lead to undertreatment of invasive aspergillosis.

## Background

Voriconazole, posaconazole and isavuconazole are triazole antifungals used to treat invasive fungal infections, including pulmonary aspergillosis. Triazoles block ergosterol synthesis by inhibiting cytochrome P450 enzymes (CYP450), leading to fungal cell death. Therapeutic drug monitoring (TDM) is important to guide the treatment of voriconazole and posaconazole as numerous factors have been associated with variability in their exposure, e.g. altered intestinal absorption and drug interactions [1].

Voriconazole has been considered as first-line treatment for invasive aspergillosis for more than a decade. Recently, posaconazole was found to be non-inferior to voriconazole when used as first-line treatment for invasive aspergillosis, with participants in the posaconazole group having fewer treatment-related adverse events than in the voriconazole group [2]. The same is true for isavuconazole, which also showed non-inferior efficacy and a significantly better safety profile, when compared to voriconazole in the same setting [3]. While the three triazoles show similar efficacy in patients with invasive aspergillosis, they have significantly different impact on liver-mediated drug metabolism [4]. Voriconazole is a substrate and a strong inhibitor of the enzymes CYP2C9, CYP2C19 and CYP3A4. In contrast, posaconazole is not metabolized to a significant extent by CYP450; it is partially metabolized via uridine glucuronosyl transferase (UGT1A4, phase 2 enzymes) [5] and is a substrate for p-glycoprotein (P-GP) efflux in vitro. It is also a strong inhibitor of P-GP [6].

Cyclosporin is a calcineurin inhibitor, used as an immunosuppressant drug, metabolized by CYP3A4. All triazoles are inhibitors of the CYP450 pathway. Therefore, triazoles strongly inhibit the metabolization of cyclosporine, leading to significantly higher plasma concentrations. It is recommended to at least halve the dose of cyclosporine when starting therapy with voriconazole or posaconazole to obtain the right exposure [4].

Flucloxacillin, a penicillin beta-lactam antibiotic, is used for infections caused by susceptible, usually Gram-positive organisms, such as *Staphylococcus aureus*. It has been described that flucloxacillin has the ability to activate the pregnane X receptor (PXR), which can induce the expression of CYP450, P-GP and UGT enzymes [7].

There is already evidence of an interaction between voriconazole and flucloxacillin, resulting in subtherapeutic plasma concentrations of voriconazole [8, 9], in the current case report, we describe an similar interaction between posaconazole and flucloxacillin.

## Case presentation

In February 2019, a 60-year-old male had a double lung transplantation for end-stage chronic obstructive pulmonary disease. Cytomegalovirus status was donor positive and recipient negative (CMV D+/R−). In June 2019, *Aspergillus fumigatus* was isolated in a bronchoalveolar lavage (BAL) culture, Aspergillus antigen detection in BAL was positive (index 6.1). On CT there was a new onset tree-in-bud pattern, consistent with probable invasive aspergillosis [10], and as a consequence, oral voriconazole (200 mg q12h after appropriate loading) was initiated five days later. Ten days after starting voriconazole, the voriconazole trough level was 2.2 mg/L, which is well within the therapeutic range (2.0–5.5 mg/L). In July, the patient presented at the emergency room with fever, shivering, headache, confusion, productive cough and vision loss of the lower-temporal quadrant of the right eye. Lab results showed an elevated C-reactive protein of 180 mg/L (ULN 5 mg/L). Chest X-ray demonstrated no consolidations. Sputum culture was negative. Broncho-alveolar lavage showed a negative Aspergillus antigen. CMV infection was excluded since PCR in serum was negative, while still being on prophylactic treatment with valganciclovir. Brain CT and MRI were negative. A lumbar puncture was culture- and PCR-negative. Ophthalmological review revealed a probable anterior ischemic opticoneuropathy (AION), no septic emboli. There was a new systolic murmur and echocardiography confirmed a new severe mitral valve regurgitation grade 4/4 with chorda rupture. According to the Duke’s criteria (1 major, 2 minor) we diagnosed a possible endocarditis. Treatment with intravenous amoxicillin, flucloxacillin 2g q4h and gentamicin was initiated. Stomatological review and PET-CT showed no focus of infection, transesophageal echocardiography showed no convincing vegetations. Blood cultures remained negative. Antibiotic therapy was given for ten days. A peripherally inserted central catheter (PICC) was placed during treatment. Four days after terminating antibiotic therapy he got a high fever and blood cultures became positive for methicillin sensitive *Staphylococcus aureus* (MSSA), due to catheter sepsis for which therapy with intravenous flucloxacillin 2g q4h was re-initiated and continued for 14 days in total. The PICC was removed.

**Fig. 1:**
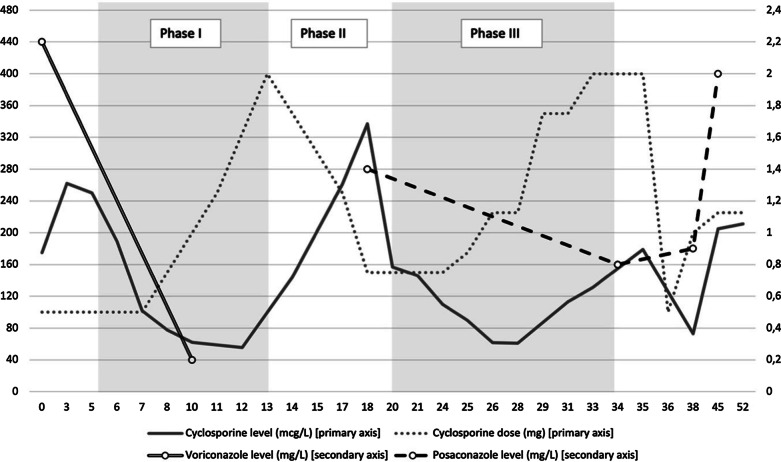
Evolution in time of cyclosporine concentrations, dose, voriconazole and posaconazole concentrations. Legend: Evolution in time (x-axis, days) of cyclosporine concentrations vs cyclosporine dose (y-axis, primary axis), voriconazole and posaconazole concentrations (y-axis, secondary axis). During phase I and III the patient was treated with flucloxacillin

The patient was immunosuppressed with cyclosporine (target trough levels 170-200 mcg/L) and oral methylprednisolone 12mg daily. During the course of the above-mentioned infectious problems, we noticed extremely varying cyclosporine, voriconazole and posaconazole concentrations. The two times that flucloxacillin was initiated, we saw an immediate drop in the concentrations of cyclosporine and both triazoles. Voriconazole was stopped because of the known possible interaction between voriconazole and flucloxacillin and was switched to oral posaconazole (300 mg q24h, tablet formulation). Interestingly an identical drop in concentrations of posaconazole was noted from 1.4 to 0.8 mg/L consistent with an interaction with flucloxacillin. At the same time, cyclosporine concentrations also decreased, from 337 to 61 mcg/L. This is summarized in Fig. 1.

The liver function was normal and there was no use of other relevant drugs (e.g., statins) during the treatment which could contribute to altered drug metabolism.

Fortunately, there were no clinical complications of the subtherapeutic plasma concentrations of the azoles and cyclosporine. The invasive pulmonary aspergillosis was cured with persistent negative Aspergillus antigen in the broncho-alveolar lavage and resolution of the radiographic abnormalities, possibly because of the already adequate treatment before the interactions started. There were no signs of acute rejection on CT scan, nor on biopsies taken at the end of the antibiotic treatment. Spirometry remained stable during the treatment and the years following, without signs of (chronic) rejection.

## Discussion and conclusions

A case report published in 2015 described that simultaneous treatment with flucloxacillin and voriconazole resulted in undetectable concentrations of voriconazole [8]. In 2017 a cohort study [9] was published where 11 of 20 patients receiving voriconazole and flucloxacillin simultaneously had significantly lowered voriconazole trough levels, defined as <1 mg/L. The median voriconazole concentration in these 11 patients during flucloxacillin treatment was 0.20 mg/L, while the median voriconazole trough level in the other 9 patients was 1.45 mg/L, thus also lower than 2 mg/L, which is often used as an alternative target trough level correlating with efficacy. The effect of flucloxacillin on plasma voriconazole concentrations was independent of the flucloxacillin dose administered. The concentrations of other known CYP3A4 substrates (such as cyclosporine and tacrolimus) in plasma were not affected during flucloxacillin therapy alone. The authors suggest that the interaction might be driven by flucloxacillin-mediated PXR activation, however they question the observed phenomenon noting that the concentrations in plasma of other drugs metabolized by CYP3A4 remained unaffected. Besides, the immediate effect of the interaction is not in agreement with the slow process of PXR induction. As mentioned above, posaconazole is not a substrate of CYP3A4 but rather of UGT1A4 and P-GP. It has been described that flucloxacillin also has the potential to induce expression of P-GP and UGT1As, mediated by PXR [11].

In Fig. 1 we show the evolution in time in our case of cyclosporine concentrations, cyclosporine dose, voriconazole and posaconazole concentrations, while the patient was treated with flucloxacillin in phase one and three. In the first phase, when we started flucloxacillin, we saw a drop in cyclosporine concentrations, which was progressive despite a gradual increase of cyclosporine dose. We concluded, based on the available literature, and on the non-measurable voriconazole concentration, that flucloxacillin lowered voriconazole concentrations thereby neutralizing the inhibiting effect of voriconazole on cyclosporine metabolism. Therefore, voriconazole was replaced by posaconazole, as such interaction was not yet described. In the second phase, after ending treatment with flucloxacillin, we saw an immediate recovery of the cyclosporine concentrations, and were able to reduce cyclosporine doses. Posaconazole trough concentrations were also within the therapeutic range (i.e., >1 mg/L) in this phase. In the third phase, when flucloxacillin was restarted, cyclosporine concentrations again decreased immediately. Simultaneously, posaconazole concentrations decreased under the threshold needed for efficacy.

To the best of our knowledge, this is the first documented interaction between posaconazole and flucloxacillin, occurring in a similar way as has been described for voriconazole. Though it has been suggested that flucloxacillin might induce CYP450, P-GP and UGT via the PXR-pathway, the underlying mechanism explaining this interaction should still be confirmed. It is accepted that the process of inducing is a slow process, in terms of days, which is not consistent with the very rapid effect after initiating and withdrawing flucloxacillin. The impact on cyclosporine exposure is considered to be a secondary effect, resulting from the decrease in azole concentrations, as it was previously shown that flucloxacillin does not induce cyclosporine nor tacrolimus concentrations [9].

It is not clear why the interaction with posaconazole has not been reported before in literature. This might be due to the fact that the combination of posaconazole and flucloxacillin is not very frequent in use, and when flucloxacillin is used, it is mostly in a short course of treatment, whereas in the meantime posaconazole concentrations might not always be monitored. A case report about post-tuberculous mycetoma describes treatment with the combination of posaconazole and flucloxacillin during one week without mentioning subtherapeutic drug concentrations [12]. Caution is warranted when combining flucloxacillin and isavuconazole; as TDM of isavuconazole is not yet implemented in many centers, the interaction might not be identified. This might lead to long term low exposure as isavuconazole has a very long half-life.

Flucloxacillin decreases plasma exposure of posaconazole, occurring in a similar way as has been described for voriconazole. We recommend close surveillance when flucloxacillin is used concurrently with posaconazole and voriconazole. Moreover, caution is warranted when combining flucloxacillin with isavuconazole, as the interaction might occur with all of the triazoles. This interaction may clearly lead to undertreatment of the invasive aspergillosis, and association of another type of antifungal drug (e.g., Amphotericin B) needs to be considered, or a switch of antibiotic treatment needs to be made. In our case, during the drops of concentration of cyclosporin, we did not increase the dose of methylprednisolone or associate any other immunosuppressant. When the subtherapeutic plasma concentrations would persist for a longer period, that surely would be an option.

Whether this interaction is also applicable for other isoxazole penicillins (e.g., dicloxacillin) is uncertain, although there has been evidence of the same PXR activation and induction of CYP3A4 by dicloxacillin, possibly meaning that it can have the same effect as flucloxacillin on drugs susceptible for this interaction [13].

## Data Availability

Not applicable.

## Abbreviations

AION: Anterior ischemic opticoneuropathy
BAL: Bronchoalveolar lavage
CMV: Cytomegalovirus
CYP450: Cytochrome P450
IPA: Invasive Pulmonary Aspergillosis
MSSA: Methicillin sensitive Staphylococcus aureus
PICC: Peripherally inserted central catheter
P-GP: P-glycoprotein
PXR: Pregnane X receptor
TDM: Therapeutic drug monitoring
UGT: Uridine glucuronosyl transferase
